# Antimicrobial resistance of *Klebsiella pneumoniae* stool isolates circulating in Kenya

**DOI:** 10.1371/journal.pone.0178880

**Published:** 2017-06-02

**Authors:** Chris Rowe Taitt, Tomasz A. Leski, Daniel P. Erwin, Elizabeth A. Odundo, Nancy C. Kipkemoi, Janet N. Ndonye, Ronald K. Kirera, Abigael N. Ombogo, Judd L. Walson, Patricia B. Pavlinac, Christine Hulseberg, Gary J. Vora

**Affiliations:** 1 Center for Bio/Molecular Science & Engineering, Naval Research Laboratory, Washington, DC United States of America; 2 US Army Medical Research Directorate-Kenya, Walter Reed Army Institute of Research, Kericho, Kenya; 3 KEMRI/US Army Medical Research Directorate-Kenya, Walter Reed Army Institute of Research, Kericho, Kenya; 4 Department of Global Health, University of Washington, Seattle, WA, United States of America; 5 Departments of Pediatrics, Medicine, and Epidemiology, University of Washington, Seattle, WA, United States of America; Ross University School of Veterinary Medicine, SAINT KITTS AND NEVIS

## Abstract

We sought to determine the genetic and phenotypic antimicrobial resistance (AMR) profiles of commensal *Klebsiella* spp. circulating in Kenya by testing human stool isolates of 87 *K*. *pneumoniae* and three *K*. *oxytoca* collected at eight locations. Over one-third of the isolates were resistant to ≥3 categories of antimicrobials and were considered multidrug-resistant (MDR). We then compared the resistance phenotype to the presence/absence of 238 AMR genes determined by a broad-spectrum microarray and PCR. Forty-six genes/gene families were identified conferring resistance to β-lactams (*ampC*/*bla*_DHA_, *bla*_CMY/LAT_, *bla*_LEN-1_, *bla*_OKP-A/OKP-B1_, *bla*_OXA-1-like_ family, *bla*_OXY-1_, *bla*_SHV_, *bla*_TEM_, *bla*_CTX-M-1_ and *bla*_CTX-M-2_ families), aminoglycosides (*aac(3)-III*, *aac(6)-Ib*, *aad*(A1/A2), *aad*(A4), *aph*(AI), *aph3/str*(A), *aph6/str*(B), and *rmtB*), macrolides (*mac*(A), *mac*(B), *mph*(A)*/mph*(K)), tetracyclines (*tet*(A), *tet*(B), *tet*(D), *tet*(G)), ansamycins (*arr*), phenicols (*catA1/cat4*, *floR*, *cmlA*, *cmr*), fluoroquinolones (*qnrS*), quaternary amines (*qacE*Δ*1*), streptothricin (*sat2*), sulfonamides (*sul1*, *sul2*, *sul3*), and diaminopyrimidines (*dfrA1*, *dfrA5*, *dfrA7*, *dfrA8*, *dfrA12*, *dfrA13/21/22/23* family, *dfrA14*, *dfrA15*, *dfrA16*, *dfrA17*). This is the first profile of genes conferring resistance to multiple categories of antimicrobial agents in western and central Kenya. The large number and wide variety of resistance genes detected suggest the presence of significant selective pressure. The presence of five or more resistance determinants in almost two-thirds of the isolates points to the need for more effective, targeted public health policies and infection control/prevention measures.

## Introduction

Antimicrobial resistance (AMR) is of significant concern in developing nations due to over-use of antimicrobial agents, widespread availability of counterfeit or substandard drugs, and poor infection control measures [1,2]. The scarcity of reliable and timely information, particularly in sub-Saharan Africa, may further limit epidemiological surveillance and effective stewardship efforts.

While only infrequently associated with diarrheal disease, *Klebsiella pneumoniae* and other klebsiellae are common intestinal commensals with significant potential to cause extraintestinal infections in severely ill patients and diarrhea in HIV/AIDS patients [3,4,5,6,7]. Of additional concern, *Klebsiella* spp. acquire, accumulate, and transfer myriad AMR determinants and therefore may represent a significant reservoir for resistance within the gut [8,9,10] and may increase the risk of resistant infections in hospital environments [5,11]. Indeed, *in vivo* transfer of AMR genes from intestinal klebsiellae to other bacterial species has been well documented [12,13,14,15,16]. Here, we use intestinal *Klebsiella* isolates collected at eight medical treatment facilities in western and central Kenya to interrogate the gut resistome and its potential for rapid evolution and spread.

## Materials and methods

### Sample collection, processing, antimicrobial susceptibility testing

Stool specimens or rectal swabs were collected into sterile, wide-mouth collection cups and aliquoted into thirds (Cary-Blair transport media, 10% formalin for parasitology, and a vial for freezing at -20C for virology) upon enrollment; previous studies showed no differences in frequency of bacterial isolation between stool samples and rectal swabs [17]. Samples were stripped of all identifiers and were assigned accession numbers before transportation to the WRAIR Microbiology Hub laboratory in Kericho (MHK) within 72 hours of collection. Samples were then plated on primary, selective, and differential media. MacConkey, MacConkey-sorbitol, sheep blood agar, Hektoen enteric agar, thiosulfate-citrate-bile-sucrose agar, cefoperazone-vancomycin-amphotericin agar, and cefsulodin-irgasan-novobiocin agar were the primary media; no specific enrichment step was performed as part of the normal workup. At 24 and 48 hours, colonies were subcultured, Gram stained, and subjected to biochemical testing (indole production, Voges-Proskauer reaction, o-nitrophenyl-Δ-D-galactopyrandoside production) before analysis on Microflex MALDI Biotyper (Bruker Daltonics, Millerica, MA, USA) and MicroScan WalkAway40 (Siemens Healthcare, Sacramento, CA, USA) systems for identification and antibiotic susceptibility testing (AST), respectively. MIC 44 and NC 66 panels were used with LabPro software updated for 2015 CLSI breakpoints [18] and automated interpretation of results. Laboratory personnel performing susceptibility testing were enrolled in External Quality Assurance/Proficiency Testing for both College of American Pathologists (three cycles/year) and United Kingdom National External Quality Assessment Service (monthly). Weekly quality control for AST was performed using recommended ATCC strains [18].

### Study sites

Samples were collected from eight Kenyan clinical sites participating in the Walter Reed Army Institute of Research (WRAIR), University of Washington/Kenya Institute of Medical Research Institute (KEMRI) collaborative research group enteric surveillance programs. These surveillance sites serve diverse communities: Mbagathi District Hospital serves a highly urban population near the center of Nairobi. The Eldoret-based clinic at Moi Barracks (MBB1) serves military service members and their families in the Kenyan highlands. Kericho District Hospital, also located in the highlands, serves a relatively rural community of tea pluckers and farmers. Kombewa is similarly considered rural. The remaining sites at the district hospitals of Kisumu, Kisii, Migori, and Homa Bay are located in western Kenya near Lake Victoria and serve both urban and rural populations largely subsistent upon agricultural and fishing economies.

### Eligibility criteria

Protocol-trained clinical staff at all sites recruited subjects experiencing acute diarrhea (three or more loose stools within a 24 hour period). The cases were recruited only from outpatient populations, and none were admitted to the hospital. Age-matched asymptomatic controls were recruited from the same sites if the subjects had not experienced acute diarrhea within the previous two week period; when possible, controls were healthy siblings close in age to the index case. Participants experiencing (chronic) diarrhea lasting more than 14 days were excluded. Medical histories were captured for a small subset of samples (*n* = 13). Both cases and controls provided basic clinical, epidemiological (water source and treatment) and demographic (age, gender, residence) information. Enrollment of all subjects required informed consent and custodial assent for subjects under 18 years of age. No diagnostic or therapeutic decisions were based on any phenotypic or genotypic data generated for this study. Work performed on this study was approved by the KEMRI and WRAIR Institutional Review Boards under KEMRI SSC #1549/WRAIR #1549 and KEMRI SSC #2056/WRAIR #1811.

### Detection of resistance determinants

The presence/absence of 238 different AMR genes was determined using the Antimicrobial Resistance Determinant Microarray (ARDM) v.2 as previously described [19,20]. Briefly, this microarray was designed for detection of >200 determinants derived from both Gram-positive and–negative bacteria. Chip content covers genes conferring resistance to 15 categories of antimicrobials (β-lactams, aminoglycosides, macrolides, lincosamides, streptogramins, quaternary amines, ansamycins, diaminopyrimidines, antimicrobial peptides, tetracyclines, phenicols, glycopeptides, platensemycin, fluoroquinolones, sulfonamides); several plasmid-borne multidrug efflux pumps are also represented on the chip. Full chip content information is given in [19]. Following sample processing, hybridization, and washing, the signal associated with each probe was determined electrochemically. An AMR gene was identified as detected when > 50% of its representative probes had signals above the mean signal from the lowest 2,128 probes + 3 standard deviations *or* when >70% of its probes had signals above either of two less stringent thresholds [20,21]. A limited set of detected AMR and integrase genes were confirmed by PCR and DNA amplicon sequencing (S1 Table).

### Statistical analysis

Statistical comparisons between populations were performed using two-tailed student's *t*-tests (assuming unequal variance). Chi-square tests were used to compare binomial proportions in independent samples (2 × *n* contingency tables). Linear regression was used to compare the number of genes/isolate with age (H_o_: slope = 0, tested by student's *t*-test).

## Results

### Sample set characteristics

A total of 90 *Klebsiella* spp. strains were isolated from participants ranging in age from 4 months to 54 years (median age 57 months). Half of the subjects presented with acute diarrheal illness and half were healthy controls. The majority of isolates came from the Kisii and Kisumu sites (37 [41.1%] and 16 [17.8%] isolates, respectively) (Table 1). Thirty-three of the isolates (36.7%) were non-susceptible to at least three categories of antimicrobials and were considered multidrug resistant (MDR) per Magiorakos [22]. One isolate, MHK02590, was considered extensively drug-resistant (non-susceptible to at least one agent in all but two or fewer antimicrobial categories; Table 2) [22]. As a whole, there were no differences between overall MDR phenotypes (*P* = 0.940) in the strains isolated from subjects with ADI and asymptomatic controls, nor between genders (*P* = 0.463). Between 80 and 90% of the tested isolates were susceptible to all β-lactams except ampicillin, to one or more aminoglycosides, and to both of the fluoroquinolones tested. Over half were susceptible to tetracycline, but more than 60% were resistant to sulfamethoxazole-trimethoprim (SXT).

**Table 1:** Summary of antimicrobial phenotypic susceptibility for diarrheal and control isolates.

Antimicrobial compound^a^	Phenotype	Case (*n* = 45)	Control (*n* = 45)	Overall (*n* = 90)
AMC	R	11 (24%)	7 (16%)	18 (20%)
	I	8 (18%)	5 (11%)	13 (14%)
	S	26 (58%)	33 (73%)	59 (66%)
SAM	R	18 (40%)	14 (31%)	32 (36%)
	I	4 (9%)	3 (7%)	7 (8%)
	S	23 (51%)	28 (62%)	51 (57%)
ATM	R	4 (9%)	4 (9%)	8 (9%)
	I	1 (2%)	-	1 (1%)
	S	40 (89%)	41 (91%)	81 (91%)
FEP	R	6 (13%)	3 (7%)	9 (10%)
	I	-	-	-
	S	39 (87%)	42 (93%)	81 (90%)
CAZ	R	1 (2%)	1 (2%)	2 (2%)
	R (ESBL)	4 (9%)	2 (4%)	6 (7%)
	I	1 (2%)	-	1 (1%)
	S	39 (87%)	42 (93%)	81 (90%)
CTX	R	1 (2%)	1 (2%)	2 (2%)
	R (ESBL)	4 (9%)	2 (4%)	6 (7%)
	I	2 (4%)	-	2 (2%)
	S	38	42 (93%)	80 (89%)
IPM	R	1 (2%)	-	1 (1%)
	I	-	3 (7%)	3 (3%)
	S	44 (98%)	42 (93%)	86 (96%)
MEM	R	-	1 (2%)	1 (1%)
	I	1 (2%)	-	1 (1%)
	S	44 (98%)	44 (98%)	88 (98%)
AMK	R	1 (2%)	1 (2%)	2 (2%)
	I	-	-	-
	S	44 (98%)	44 (98%)	88 (98%)
GEN	R	3 (7%)	4 (9%)	7 (8%)
	I	1 (2%)	1 (2%)	2 (2%)
	S	41 (91%)	40 (89%)	81 (90%)
TOB	R	3 (7%)	2 (4%)	5 (6%)
	I	-	2 (4%)	2 (2%)
	S	42 (93%)	41 (91%)	83 (92%)
TET	R	18 (40%)	15 (33%)	33 (37%)
	I	5 (11%)	3 (7%)	8 (9%)
	S	22 (449%)	27 (60%)	49 (54%)
CIP	R	1 (2%)	-	1 (1%)
	I	1 (2%)	-	1 (1%)
	S	43 (96%)	45 (100%)	88 (98%)
LVX	R	2 (4%)	-	2 (2%)
	I	-	1 (2%)	1 (1%)
	S	43 (96%)	44 (98%)	87 (97%)
SXT	R	25 (56%)	30 (67%)	55 (61%)
	I	-	-	-
	S	20 (44%)	15 (33%)	35 (39%)

^a^Antimicrobial compounds are grouped together according to categories used to define MDR per Magiorakos [22]. AMC–amoxicillin/clavulanate; SAM–ampicillin/sulbactam; ATM–aztreonam; FEP–cefepime; CAZ–ceftazidime; CTX–cefotaxime; IMP–imipenem; MEM–meropenem; AMK–amikacin; GEN–gentamicin; TOB–tobramycin; TET–tetracycline; CIP–ciprofloxacin; LVX–levofloxacin; SXT–trimethoprim/sulfamethoxazole; S–sensitive; I–intermediate; R–resistant. ESBL–Extended-spectrum β-lactamase

**Table 2:** Metadata and phenotypic antimicrobial susceptibility for individual isolates.

Strain no.	age		date isolated	site^b^	Antimicrobial compound^a^															Ctrl/Cs^c^
		gender			AMC	SAM	ATM	FEP	CAZ	CTX	IPM	MEM	AMK	GEN	TOB	TET	CIP	LVX	SXT	
MHK00504	11m	F	7/10/2010	Ku	R	R	S	S	S	S	I	S	S	S	S	R	S	S	R	ctrl
MHK01305	18yr 6m	M	5/24/2011	Ki	S	R	S	S	S	S	S	S	S	S	S	R	S	S	R	cs
MHK01419	3yr 2m	F	6/21/2011	Ki	S	R	S	S	S	S	S	S	S	R	I	R	S	S	R	ctrl
MHK01814	9m	M	9/28/2011	Mb	R	R	S	S	I	S	R	S	S	S	S	R	S	S	R	cs
MHK02123^d^	21yr	F	1/11/2012	Ki	S	I	S	S	S	S	S	S	S	S	S	R	S	S	R	ctrl
MHK02126^d^	1yr	M	1/11/2012	Ki	S	S	S	S	S	S	S	S	S	S	S	S	S	S	R	ctrl
MHK02178	21yr	M	1/21/2012	Ki	R	R	S	S	S	S	I	S	S	S	S	R	S	S	R	ctrl
MHK02303	2yr 1m	F	2/11/2012	Ki	R	R	S	S	S	S	S	S	S	R	I	R	S	S	R	ctrl
MHK02499	1yr 3m	M	3/29/2012	Ki	I	R	R	R	ESBL	ESBL	S	S	S	I	S	S	S	S	R	ctrl
MHK02590	6m	M	4/14/2012	Mb	R	R	R	R	R	R	S	S	R	R	R	R	R	R	R	cs
MHK02631	54yr	F	4/20/2012	Ki	S	S	S	S	S	S	S	S	S	S	S	R	S	S	R	ctrl
MHK02678	1yr 10m	F	5/1/2012	Ki	S	S	S	S	S	S	S	S	S	S	S	S	S	S	R	ctrl
MHK02690	9m	M	5/4/2012	Mb	R	R	S	S	S	S	S	S	S	S	S	I	S	S	R	cs
MHK02780	4m	F	5/29/2012	Ki	S	S	S	S	S	S	S	S	S	S	S	R	S	S	S	cs
MHK03026	8yr	F	7/13/2012	Ku	I	R	S	S	S	S	S	S	S	S	S	R	S	S	R	cs
MHK04212	11m	M	11/15/2013	M1	R	S	S	S	S	S	S	S	S	S	S	S	S	S	R	cs
MHK04617	5m	M	11/16/2013	Mb	I	R	S	S	S	S	S	S	S	S	S	S	S	S	R	cs
MHK04622	8m	M	11/20/2013	Ki	S	S	S	S	S	S	S	S	S	S	S	S	S	S	S	cs
MHK04775	2yr 1m	F	2/1/2014	Ku	S	S	S	S	S	S	S	S	S	S	S	S	S	S	R	ctrl
MHK04776	2yr 6m	F	2/1/2014	Ku	I	R	S	S	S	S	S	S	S	S	S	S	S	S	R	cs
MHK04777	51yr	M	2/1/2014	Ko	S	R	S	S	S	S	S	S	S	S	S	R	S	S	R	ctrl
MHK04779	2yr 3m	M	2/4/2014	Ke	S	S	S	S	S	S	S	S	S	S	S	S	S	S	S	cs
MHK04786	4yr	M	2/5/2014	Ki	I	I	S	S	S	S	S	S	S	S	S	R	S	S	R	cs
MHK04792	43yr	M	2/5/2014	Ke	S	S	S	S	S	S	S	S	S	S	S	R	S	S	S	ctrl
MHK04804	3yr 9m	F	2/7/2014	Ki	S	R	S	S	S	S	S	S	S	S	S	R	S	S	R	ctrl
MHK04812	3yr	M	3/27/2014	Ke	R	R	S	R	ESBL	ESBL	S	S	S	S	S	S	S	S	R	cs
MHK04813	1yr 2m	M	3/28/2014	Ku	I	R	S	S	S	S	S	S	S	S	S	S	S	S	R	cs
MHK04819	1yr 4m	M	4/1/2014	Ku	S	S	S	S	S	S	S	S	S	S	S	S	S	S	R	ctrl
MHK04821	2yr 3m	F	4/2/2014	Ke	S	S	S	S	S	S	S	S	S	S	S	S	S	S	S	cs
MHK04822	2yr 11m	M	4/3/2014	Ki	S	R	S	S	S	S	S	S	S	S	S	R	S	S	R	ctrl
MHK04834	19yr	M	4/9/2014	Ko	S	S	S	S	S	S	S	S	S	S	S	I	S	S	S	ctrl
MHK04838	4yr	F	4/10/2014	Ku	S	S	S	S	S	S	S	S	S	S	S	S	S	S	S	cs
MHK04847	28yr	F	4/11/2014	Ko	S	S	S	S	S	S	S	S	S	S	S	R	S	S	S	cs
MHK04864	32yr	F	4/17/2014	Ki	S	S	S	S	S	S	S	S	S	S	S	I	S	S	S	ctrl
MHK04872	22yr	F	4/18/2014	Ku	I	R	S	S	S	S	S	S	S	S	S	R	S	S	R	cs
MHK04885	2yr 5m	M	4/24/2014	Ke	S	S	S	S	S	S	S	S	S	S	S	R	S	S	R	cs
MHK04900	3yr 5m	F	4/28/2014	Ke	S	S	S	S	S	S	S	S	S	S	S	S	S	S	S	ctrl
MHK04904	22yr	F	4/29/2014	Ki	S	I	S	S	S	S	S	S	S	I	S	S	S	S	R	cs
MHK04908	1yr 3m	M	4/30/2014	Ku	S	R	S	S	S	S	S	S	S	S	S	R	S	S	R	cs
MHK04919	3yr	M	5/6/2014	Ki	I	R	S	S	S	S	S	S	S	S	S	S	S	S	R	ctrl
MHK04922	3yr	F	5/7/2014	Ki	R	R	R	R	R	R	I	S	R	R	R	S	S	I	R	ctrl
MHK04923	24yr	M	5/7/2014	Ki	S	S	S	S	S	S	S	R	S	S	S	S	S	S	R	ctrl
MHK04926	31yr	F	5/7/2014	Ku	S	S	S	S	S	S	S	S	S	S	S	S	S	S	R	ctrl
MHK04928	5yr	F	5/9/2014	Ki	S	S	S	S	S	S	S	S	S	S	S	S	S	S	S	ctrl
MHK04930	28yr	M	5/9/2014	M1	R	R	R	R	ESBL	ESBL	S	S	S	R	R	S	S	S	R	ctrl
MHK04941	1yr 8m	M	5/14/2014	M1	R	S	S	S	S	S	S	S	S	S	S	R	S	S	R	ctrl
MHK04943	28yr	F	5/15/2014	Ki	S	S	S	S	S	S	S	S	S	S	S	S	S	S	S	cs
MHK04946	17yr	F	5/16/2014	Ki	S	S	S	S	S	S	S	S	S	S	S	S	S	S	S	cs
MHK04947	36yr	F	5/16/2014	Ko	R	R	S	S	S	S	S	S	S	S	S	I	S	S	R	cs
MHK04948	38yr	M	5/16/2014	Ko	S	S	S	S	S	S	S	S	S	S	S	S	S	S	S	ctrl
MHK04957	37yr	F	5/17/2014	M1	S	S	S	S	S	I	S	S	S	S	S	I	S	S	S	cs
MHK04960	8m	M	5/20/2014	Ki	S	S	S	S	S	S	S	S	S	S	S	S	S	S	S	ctrl
MHK04967	15yr	M	5/22/2014	M1	I	I	S	S	S	S	S	S	S	S	S	R	S	S	R	ctrl
MHK04980	30yr	M	5/23/2014	M1	I	I	S	S	S	S	S	S	S	S	S	R	S	S	R	cs
MHK04983	1yr 1m	M	5/24/2014	Ku	I	R	S	S	S	S	S	S	S	S	S	R	S	S	R	ctrl
MHK04984	6m	M	5/24/2014	Ku	S	S	S	S	S	S	S	S	S	S	S	S	S	S	R	ctrl
MHK05010	8yr	F	5/31/2014	Ki	I	R	R	S	S	S	S	S	S	S	S	S	S	S	R	ctrl
MHK05013a	35yr	F	5/31/2014	M1	S	S	S	S	S	S	S	S	S	S	S	S	S	S	R	ctrl
MHK05013b	35yr	F	5/31/2014	M1	S	S	S	S	S	S	S	S	S	S	S	S	S	S	R	ctrl
MHK05014a	32yr	M	5/31/2014	M1	S	S	S	S	S	S	S	S	S	S	S	S	S	S	S	ctrl
MHK05014b^d^	32yr	M	5/31/2014	M1	S	S	S	S	S	S	S	S	S	S	S	S	S	S	S	ctrl
MHK05017	52yr	F	6/5/2014	Ko	S	S	S	S	S	S	S	S	S	S	S	S	S	S	R	cs
MHK05018	32yr	M	6/5/2014	M1	S	S	S	S	S	S	S	S	S	S	S	S	S	S	S	ctrl
MHK05018-1b	32yr	M	6/5/2014	M1	S	S	S	S	S	S	S	S	S	S	S	S	S	S	S	ctrl
MHK05021	7yr	M	6/5/2014	Ki	S	R	S	S	S	S	S	S	S	S	S	S	S	S	R	ctrl
MHK05027	7yr	M	6/6/2014	Ki	R	R	S	S	S	S	S	S	S	S	S	R	S	S	R	cs
MHK05028	5yr	F	6/6/2014	Ki	S	S	S	S	S	S	S	S	S	S	S	R	S	S	R	cs
MHK05042	2yr 9m	M	6/11/2014	Ki	R	I	S	S	S	S	S	S	S	S	S	R	S	S	R	ctrl
MHK05046	4yr 10m	M	6/12/2014	Ki	S	S	S	S	S	I	S	S	S	S	S	S	S	S	S	cs
MHK05068	6yr	M	6/20/2014	Ki	R	R	R	R	ESBL	ESBL	S	S	S	R	R	S	I	S	R	cs
MHK05070	1yr	F	6/21/2014	Ku	S	S	S	S	S	S	S	S	S	S	S	R	S	S	R	ctrl
MHK05072	4yr 6m	F	6/21/2014	Ki	S	S	S	S	S	S	S	S	S	S	S	S	S	S	S	cs
MHK05080	9yr	M	6/27/2015	Ki	S	S	S	S	S	S	S	S	S	S	S	I	S	S	S	cs
MHK05084	5yr	F	6/28/2014	Ku	R	I	S	S	S	S	S	S	S	S	S	R	S	S	S	cs
MHK05090	4yr 6m	M	7/2/2014	Ku	S	S	S	S	S	S	S	S	S	S	S	S	S	S	S	ctrl
MHK05091	5yr 10m	M	7/2/2014	Ku	R	R	R	R	ESBL	ESBL	S	S	S	S	S	R	S	S	R	cs
MHK05094	23yr	F	7/4/2014	Ki	S	S	S	S	S	S	S	S	S	S	S	S	S	S	S	ctrl
NTS01697	4yr 1m	M	6/12/2014	Ki	R	R	R	R	ESBL	ESBL	S	I	S	R	R	S	S	R	R	cs
NTS01699	5yr 5m	M	6/12/2014	Mg	S	S	S	S	S	S	S	S	S	S	S	I	S	S	S	cs
NTS01703	2yr 8m	F	6/13/2014	Hy	S	S	S	S	S	S	S	S	S	S	S	R	S	S	R	cs
NTS01705	7m	F	6/13/2014	Hy	S	R	S	R	S	S	S	S	S	S	S	S	S	S	R	cs
NTS01707	4yr 9m	F	6/13/2014	Mg	S	S	S	S	S	S	S	S	S	S	S	R	S	S	S	ctrl
NTS01708	2yr 9m	M	6/14/2014	Ki	S	S	S	S	S	S	S	S	S	S	S	S	S	S	S	cs
NTS01732	2yr 10m	F	6/25/2014	Hy	S	S	S	S	S	S	S	S	S	S	S	S	S	S	S	cs
NTS01745	11m	F	7/2/2014	Ki	S	S	S	S	S	S	S	S	S	S	S	S	S	S	S	cs
NTS01747	5yr	M	7/3/2014	Hy	S	S	S	S	S	S	S	S	S	S	S	S	S	S	R	ctrl
NTS01749	4yr 3m	M	7/3/2014	Mg	S	S	S	S	S	S	S	S	S	S	S	I	S	S	S	cs
NTS01755	3yr 1m	F	7/5/2014	Ki	I	R	S	S	S	S	S	S	S	S	S	R	S	S	R	cs
NTS01793	3yr 2m	M	8/2/2014	Hy	S	S	I	S	S	S	S	S	S	S	S	S	S	S	S	cs
NTS01936	5yr 6m	M	6/26/2014	Mg	S	S	S	S	S	S	S	S	S	S	S	S	S	S	S	ctrl

^a^Antimicrobial compounds are grouped together according to categories used to define MDR per Magiorakos [22]. AMC–amoxicillin/clavulanate; SAM–ampicillin/sulbactam; ATM–aztreonam; FEP–cefepime; CAZ–ceftazidime; CTX–cefotaxime; IMP–imipenem; MEM–meropenem; AMK–amikacin; GEN–gentamicin; TOB–tobramycin; TET–tetracycline; CIP–ciprofloxacin; LVX–levofloxacin; SXT–trimethoprim/sulfamethoxazole; S–sensitive; I–intermediate; R–resistant. ESBL–Extended-spectrum β-lactamase

^b^Collection site: Hy–Homabay; Ke–Kericho; Ki–Kisii; Ko–Kombewa; Ku–Kisumu; Mb–Mbagathi; Mg–Migori; M1 –Moi Barracks at Eldoret

^c^ctrl–healthy control; cs–case of acute diarrheal illness

^d^*K*. *oxytoca*

A total of 46 AMR genes or gene families covering 11 categories of antimicrobials were identified amongst the 90 isolates using a broad-range microarray (Table 3). PCR was used to verify the presence of a select group of genes detected by microarray, as well as ancillary genes associated with specific combinations of AMR determinants (S1 Table). All but six isolates harbored multiple resistance determinants (Table 4). While there were no differences in MDR phenotype between age quartiles (*P* = 0.336), a small but significant inverse relationship was observed between the total number of genes per isolate and age (*P* = 0.029; *t*-test of linear regression), with isolates from younger subjects harboring a larger number of genes. No significant differences in genes/isolate were observed between diarrheal and control isolates (*P* = 0.458) or between genders (*P* = 0.184). The disparate numbers of isolates collected at the various sites (*n* = 4 to *n* = 37) precluded any statistically valid site-to-site comparisons. However, sites with highest percentages of MDR phenotype, Mbagathi (3 of 4 isolates) and Kisii (17 of 37 isolates), also harbored the widest overall varieties of resistance determinants (28 and 41 determinants, respectively).

**Table 3:** Summary of AMR genes in the tested population.

gene	case (*n* = 45)	control (*n* = 45)	overall (*n* = 90)
β-lactams			
*ampC*/*bla*_DHA_	0 (0%)	1 (2%)	1 (1%)
*bla*_CMY/LAT_ family	1 (2%)	0 (0%)	1 (1%)
*bla*_LEN-1_	32 (71%)	29 (64%)	61 (68%)
*bla*_OKP-A/OKP-B1_	5 (11%)	5 (11%)	10 (11%)
*bla*_OXA-1_	3 (7%)	0 (0%)	3 (3%)
*bla*_OXY-1_	0 (0%)	4 (9%)	4 (4%)
*bla*_SHV_ family	43 (95%)	35 (77%)	78 (87%)
*bla*_TEM_ family	29 (64%)	23 (51%)	52 (58%)
*bla*_CTX-M-1_ family	5 (11%)	3 (7%)	8 (9%)
*bla*_CTX-M-2_ family	1 (2%)	0 (0%)	1 (1%)
aminoglycosides			
*aac(3)-III*	3 (7%)	2 (4%)	5 (6%)
*aac(6)-Ib*	3 (7%)	1 (2%)	4 (4%)
*aad*(A1/A2) family	10 (22%)	8 (18%)	18 (20%)
*aad*(A4)	1 (2%)	0 (0%)	1 (1%)
*aph*(AI)	3 (7%)	4 (9%)	7 (8%)
*aph3/str*(A)	23 (51%)	21 (47%)	44 (49%)
*aph6/str*(B)	25 (56%)	22 (49%)	47 (52%)
*rmtB*	0 (0%)	1 (2%)	1 (1%)
macrolides			
*mac*(A)	16 (39%)	13 (29%)	29 (32%)
*mac*(B)	13 (29%)	12 (27%)	25 (28%)
*mph*(A)/*mph*(K) family	4 (9%)	2 (4%)	6 (7%)
tetracyclines			
*tet*(A)	7 (16%)	9 (20%)	16 (18%)
*tet*(B)	4 (9%)	5 (11%)	9 (10%)
*tet*(D)	6 (13%)	5 (11%)	11 (12%)
*tet*(G)	0 (0%)	1 (2%)	1 (1%)
ansamycins			
*arr*	1 (2%)	1 (2%)	2 (2%)
phenicols			
*catA1/cat4* family	7 (16%)	2 (4%)	7 (8%)
*floR*	1 (2%)	0 (0%)	1 (1%)
*cmlA*	1 (2%)	0 (0%)	1 (1%)
*cmr*	6 (13%)	14 (31%)	32 (36%)
fluoroquinolones			
*qnrS*	2 (4%)	0 (0%)	2 (2%)
quaternary amines			
*qacEΔ1*	17 (38%)	11 (24%)	28 (31%)
streptothricin			
*sat2*	2 (4%)	2 (4%)	4 (4%)
sulfonamides			
*sul1*	17 (38%)	11 (24%)	28 (31%)
*sul2*	25 (56%)	22 (49%)	
*sul3*	1 (2%)	0 (0%)	1 (1%)
diaminopyrimidines			
*dfrA1*	6 (13%)	3 (7%)	9 (10%)
*dfrA12*	1 (2%)	2 (4%)	3 (3%)
*dfrA13/21/22/23* family	1 (2%)	0 (0%)	1 (1%)
*dfrA14*	8 (18%)	10 (22%)	18 (20%)
*dfrA15*	2 (4%)	1 (2%)	3 (3%)
*dfrA16*	0 (0%)	2 (4%)	2 (2%)
*dfrA17*	1 (2%)	0 (0%)	1 (1%)
*dfrA5*	3 (7%)	4 (9%)	7 (8%)
*dfrA7*	4 (9%)	1 (2%)	5 (6%)
*dfrA8*	3 (7%)	2 (4%)	5 (6%)

**Table 4:** AMR genes present in individual Kenyan *Klebsiella* spp. isolates.

strain no.	Resistance determinant(s)									
	β-Lactams	Aminoglycosides	Macrolides	Tetra-cyclines	Ansa-mysin	Phenicol	Quino-lones	Quaternary amines, strepto-thricin	Sulfon-amide	Diamino-pyrimidine
MHK00504	*bla*_LEN_, ***bla***_**SHV**_,‡ *bla*_TEM_	*aad*(A1/A2), *aph3/str*(A), *aph6/str*(B)	*mac*(A), *ma*c(B)	*tet*(B)		*cmr*		*sat2*	*sul2*	*dfrA1*
MHK01305	*bla*_OXA-1-like_, *bla*_TEM_, ***bla***_**CTX-M-1**_ **family**, (*bla*_SHV_)	*aac(6)-Ib*, *aph3/str*(A), *aph6/str*(B)	*mac*(A), *ma*c(B)	*tet*(B)	*arr*	*catA1/cat4*, *cmr*		*qacEΔ1*	*sul1*, *sul2*	*dfrA14*
MHK01419	*bla*_TEM_, (*bla*_SHV_)	*aph3/str*(A), *aph6/str*(B)	*mac*(A)	*tet*(B)					*sul2*	*dfrA8*
MHK01814	*bla*_LEN_, *bla*_SHV_, *bla*_TEM_	*aph3/str*(A), *aph6/str*(B)	*mac*(A), *ma*c(B)	*tet*(D)		*cmr*		*qacEΔ1*	*sul1*, *sul2*	*dfrA14*, *dfrA7*
MHK02123	*bla*_OXY-1_	*aph*(AI)				*cmr*				
MHK02126	*bla*_OXY-1_	*aph*(AI)								
MHK02178		*aad*(A1/A2), *aph3/str*(A), *aph6/str*(B)	*mac*(A), *ma*c(B)	*tet*(A)		*cmr*		*sat2*	*sul2*	*dfrA14*
MHK02303	*bla*_LEN_, ***bla***_**SHV**_		*mac*(A), *ma*c(B)			*cmr*				
MHK02499	*bla*_TEM_, ***bla***_**CTX-M-1**_ **family**	*aph3/str*(A), *aph6/str*(B)	*mac*(A), *ma*c(B)	*tet*(A)		*cmr*			*sul2*	
MHK02590	*bla*_LEN_, *bla*_OXA-1-like,_ *bla*_SHV_, *bla*_TEM_, ***bla***_**CTX-M-1**_ **family**	*aac(3)-III*, *aac(6)-Ib*, *aad*(A1/A2), *aph3/str*(A), *aph6/str*(B)	*mac*(A), *ma*c(B), *mph*(A)/*mph*(K)	*tet*(A)		*catA1/cat4*, *cmlA*, *cmr*			*sul2*, *sul3*	*dfrA12*, *dfrA14*
MHK02631	*bla*_LEN_, ***bla***_**SHV**_, *bla*_TEM_	*aph3/str*(A), *aph6/str*(B)	*mac*(A), *ma*c(B)			*cmr*			*sul2*	*dfrA14*
MHK02678	*bla*_TEM_, (*bla*_SHV_)	*aph3/str*(A), *aph6/str*(B)	*mac*(A), *ma*c(B)	*tet*(B)		*cmr*			*sul2*	*dfrA8*
MHK02690	*bla*_TEM_, (*bla*_SHV_)	*aad*(A1/A2), *aph6/str*(B)	*mac*(A), *ma*c(B)	*tet*(D)		*cmr*		*qacEΔ1*, *sat2*	*sul1*, *sul2*	*dfrA1*
MHK02780	***bla***_**SHV**_		*mac*(A), *ma*c(B)			*cmr*				
MHK03026	*bla*_LEN_, ***bla***_**SHV**_, *bla*_TEM_	*aad*(A4), *aph3/str*(A), *aph6/str*(B)		*tet*(D)		*catA1/cat4*			*sul2*	*dfrA13/21/22/23* family
MHK04212	*bla*_LEN_, (*bla*_SHV_)	*aph*(AI)				*cmr*				
MHK04617	*bla*_LEN_, ***bla***_**SHV**_, *bla*_TEM_							*qacEΔ1*	*sul1*	*dfrA5*
MHK04622	*bla*_LEN_, ***bla***_**SHV**_									
MHK04775	*bla*_LEN_, ***bla***_**SHV**_	*aph3/str*(A), *aph6/str*(B)							*sul2*	*dfrA14*
MHK04776	*bla*_LEN_, ***bla***_**SHV**_	*aph3/str*(A), *aph6/str*(B)						*qacEΔ1*	*sul1*, *sul2*	*dfrA7*
MHK04777	*bla*_LEN_, *bla*_SHV_, *bla*_TEM_	*aph3/str*(A), *aph6/str*(B)		*tet*(D)				*qacEΔ1*	*sul1*, *sul2*	*dfrA5*
MHK04779	*bla*_LEN_, ***bla***_**SHV**_									
MHK04786	*bla*_LEN_, *bla*_OKP-A/-B_, (*bla*_SHV_), *bla*_TEM_									
MHK04792	*bla*_LEN_, *bla*_OKP-A/-B_, ***bla***_**SHV**_, *bla*_TEM_	*aph6/str*(B)								
MHK04804	*bla*_LEN_, *bla*_SHV_, *bla*_TEM_	*aph3/str*(A), *aph6/str*(B)		*tet*(D)				*qacEΔ1*	*sul1*, *sul2*	*dfrA5*
MHK04812	*bla*_CMY/LAT_, *bla*_LEN_, *bla*_TEM_, (*bla*_SHV_)	*aad*(A1/A2), *aph*(AI), *aph3/str*(A), *aph6/str*(B)	*mac*(A), *ma*c(B)			*cmr*		*qacEΔ1*	*sul1*, *sul2*	
MHK04813	*bla*_TEM_, (*bla*_SHV_)	*aph3/str*(A), *aph6/str*(B)	*mac*(A), *ma*c(B)			*cmr*			*sul2*	*dfrA8*
MHK04819	*bla*_LEN_, ***bla***_**SHV**_, *bla*_TEM_	*aad*(A1/A2)		*tet*(D)				*qacEΔ1*	*sul2*	*dfrA16*, *dfrA5*
MHK04821	(*bla*_SHV_)							*qacEΔ1*	*sul1*	
MHK04822	*bla*_LEN_, ***bla***_**SH**V_, *bla*_TEM_	*aph*(AI), *aph3/str*(A), *aph6/str*(B)		*tet*(D)					*sul1*, *sul2*	*dfrA14*
MHK04834	***bla***_**SHV**_									
MHK04838	*bla*_LEN_, *bla*_OKP-A/-B_, ***bla***_**SHV**_, *bla*_TEM_									
MHK04847	*bla*_LEN_, *bla*_OKP-A/-B_, ***bla***_**SHV**_, *bla*_TEM_					*cmr*				
MHK04864	*bla*_LEN_, (*bla*_SHV_)									
MHK04872	*bla*_LEN_, *bla*_SHV_, *bla*_TEM_			*tet*(D)				*qacEΔ1*	*sul1*, *sul2*	*dfrA5*
MHK04885	*bla*_LEN_, ***bla***_**SHV**_, *bla*_TEM_	*aad*(A1/A2)		*tet*(A)				*qacEΔ1*	*sul1*	*dfrA1*
MHK04900	*bla*_LEN_, ***bla***_**SHV**_, *bla*_TEM_		*mac*(A), *ma*c(B)			*cmr*				
MHK04904	*bla*_LEN_, ***bla***_**SHV**_									
MHK04908	*bla*_LEN_, ***bla***_**SHV**_, *bla*_TEM_			*tet*(D)				*qacEΔ1*	*sul1*, *sul2*	*dfrA5*
MHK04919	*bla*_LEN_, *bla*_SHV_, *bla*_TEM_	*aph3/str*(A), *aph6/str*(B)	*mac*(A), *ma*c(B)			*cmr*			*sul2*	*dfrA14*
MHK04922	*ampC*/*bla*_DHA_, *bla*_LEN_, ***bla***_**SHV**_, *bla*_TEM_, ***bla***_**CTX-M-1**_ **family**	*aac(3)-III*, *aac(6)-Ib*, *aph*(AI), *aph3/str*(A), *aph6/str*(B), *rmtB*,		*tet*(A), *tet*(G)	*arr*			*qacEΔ1*	*sul1*, *sul2*	*dfrA12*
MHK04923	*bla*_LEN_, ***bla***_**SHV**_, *bla*_TEM_									
MHK04926	*bla*_LEN_, ***bla***_**SHV**_									
MHK04928	*bla*_LEN_, ***bla***_**SHV**_									
MHK04930	*bla*_SHV_, *bla*_TEM_, ***bla***_**CTX-M-1**_ **family**	*aac(3)-III*, *aad*(A1/A2)						*qacEΔ1*	*sul1*	*dfrA12*
MHK04941	***bla***_**SHV**_	*aph3/str*(A), *aph6/str*(B)		*tet*(A)					*sul2*	*dfrA14*
MHK04943	*bla*_LEN_, ***bla***_**SHV**_, *bla*_TEM_									
MHK04946	*bla*_LEN_									
MHK04947	*bla*_TEM_, (bla_SHV_)	*aph3/str*(A), *aph6/str*(B)	*mac*(A), *mph*(A)/*mph*(K)			*cmr*			*sul2*	
MHK04948	*bla*_LEN_, ***bla***_**SHV**_									
MHK04957	***bla***_**SHV**_									
MHK04960	*bla*_LEN_, ***bla***_**SHV**_									
MHK04967	*bla*_SHV_, *bla*_TEM_	*aph3/str*(A), *aph6/str*(B)		*tet*(A)		*catA1/cat4*		*qacEΔ1*	*sul1*, *sul2*	*dfrA7*
MHK04980	*bla*_LEN_, ***bla***_**SHV**_, *bla*_TEM_	*aph3/str*(A), *aph6/str*(B)	*mph*(A)/*mph*(K)						*sul2*	*dfrA14*
MHK04983	*bla*_OKP-A/-B_, *bla*_TEM_	*aad*(A1/A2)		*tet*(A)				*qacEΔ1*	*sul1*	*dfrA16*
MHK04984	***bla***_**SHV**_	*aph3/str*(A), *aph6/str*(B)							*sul2*	*dfrA14*
MHK05010	*bla*_LEN_, ***bla***_**SHV**_, *bla*_TEM_	*aph3/str*(A), *aph6/str*(B)	*mac*(A), *ma*c(B) *mph*(A)/*mph*(K)	*tet*(B)		*catA1/cat4*, *cmr*			*sul2*	*dfrA14*
MHK05013a	*bla*_LEN_, *bla*_SHV_, *bla*_TEM_	*aph3/str*(A), *aph6/str*(B)	*mph*(A)/*mph*(K)						*sul2*	*dfrA14*
MHK05013b	*bla*_LEN_, *bla*_OKP-A/-B_, (*bla*_SHV_), *bla*_TEM_	*aad*(A1/A2)						*qacEΔ1*	*sul1*	*dfrA15*
MHK05014a	*bla*_OXY-1_									
MHK05014b	*bla*_OXY-1,_ *bla*_TEM_									
MHK05017	*bla*_LEN_, ***bla***_**SHV**_	*aph3/str*(A), *aph6/str*(B)		*tet*(A)					*sul2*	
MHK05018	*bla*_LEN_					*cmr*				
MHK05018-1B	*bla*_LEN_, *bla*_SHV_		*mac*(A), *ma*c(B)			*cmr*			*sul2*	
MHK05021	*bla*_LEN_, *bla*_SHV_, *bla*_TEM_	*aad*(A1/A2), *aph3/str*(A), *aph6/str*(B)	*mac*(A), *ma*c(B)	*tet*(A)		*cmr*		*qacEΔ1*	*sul1*, *sul2*	*dfrA1*
MHK05027	*bla*_LEN_, ***bla***_**SHV**_, *bla*_TEM_	*aph3/str*(A), *aph6/str*(B)	*mac*(A), *ma*c(B)	*tet*(A), *tet*(B)		*cmr*			*sul2*	*dfrA14*
MHK05028	*bla*_LEN_, ***bla***_**SHV**_, *bla*_TEM_	*aad*(A1/A2), *aph3/str*(A), *aph6/str*(B)		*tet*(A)				*qacEΔ1*	*sul1*, *sul2*	*dfrA1*
MHK05042	*bla*_LEN_, ***bla***_**SH**V_, *bla*_TEM_	*aph3/str*(A), *aph6/str*(B)		*tet*(A), *tet*(D)				*qacEΔ1*	*sul1*, *sul2*	*dfrA5*
MHK05046	*bla*_SHV_	*aph3/str*(A), *aph6/str*(B)								
MHK05068	*bla*_LEN_, *bla*_OXA-1-like_, (*bla*_SHV_), *bla*_TEM_, ***bla***_**CTX-M-1**_ **family**	*aac(3)-III*, *aac(6)-Ib* family, *aph*(AI), *aph3/str*(A), *aph6/str*(B)	*mph*(A)/*mph*(K)				*qnrS*	*qacEΔ1*	*sul1*, *sul2*	*dfrA15*, *dfrA17*
MHK05070	*bla*_OKP-A/-B_	*aad*(A1/A2)		*tet*(A)				*qacEΔ1*	*sul1*	*dfrA1*
MHK05072	*bla*_LEN_, ***bla***_**SHV**_, *bla*_TEM_	*aad*(A1/A2), *aph3/str*(A), *aph6/str*(B)	*mac*(A), *ma*c(B)	*tet*(B)		*cmr*	*qnrS*	*qacEΔ1*	*sul1*, *sul2*	*dfrA1*, *dfrA14*
MHK05080	*bla*_LEN_, ***bla***_**SHV**_									
MHK05084	*bla*_OKP-A/-B_, *bla*_TEM_	*aph3/str*(A), *aph6/str*(B)	*mac*(A), *ma*c(B)	*tet*(B)		*cmr*			*sul2*	*dfrA8*
MHK05090	*bla*_OKP-A/-B_	*aph3/str*(A), *aph6/str*(B)								
MHK05091	***bla***_**SHV**_, *bla*_TEM_, ***bla***_**CTX-M-1**_ **family**	*aad*(A1/A2), *aph3/str*(A), *aph6/str*(B)	*mac*(A), *ma*c(B)	*tet*(A)		*catA1/cat4 floR*, *cmr*		*qacEΔ1*	*sul1*, *sul2*	*dfrA14*, *dfrA15*
MHK05094	*bla*_LEN_, (*bla*_SHV_)									
NTS01697	***bla***_**SHV**_, *bla*_TEM_, ***bla***_**CTX-M-1**_ **family**	*aac(3)-III*, *aph3/str*(A), *aph6/str*(B)							*sul2*	*dfrA14*
NTS01699	*bla*_LEN_, ***bla***_**SHV**_									
NTS01703	***bla***_**SHV**_	*aad*(A1/A2)						*sat2*		*dfrA1*
NTS01705	*bla*_LEN_, ***bla***_**SHV**_, *bla*_TEM_	*aph3/str*(A), *aph6/str*(B)	*mac*(A)			*catA1/cat4 cmr*		*qacEΔ1*	*sul1*, *sul2*	*dfrA7*
NTS01707	*bla*_LEN_, *bla*_SHV_									
NTS01708	*bla*_LEN_, *bla*_OKP-A/-B_, ***bla***_**SHV**_, *bla*_TEM_, *bla*_CTX-M-2_ family									
NTS01732	*bla*_LEN_, ***bla***_**SHV**_	*aph3/str*(A), *aph6/str*(B)							*sul2*	
NTS01745	*bla*_LEN_, ***bla***_**SHV**_, *bla*_TEM_		*mac*(A)			*cmr*				
NTS01747	*bla*_LEN_, (*bla*_SHV_)	*aph3/str*(A), *aph6/str*(B)	*mac*(A), *ma*c(B)	*tet*(B)		*cmr*			*sul2*	*dfrA14*
NTS01749	*bla*_LEN_, ***bla***_**SHV**_, *bla*_TEM_	*aad*(A1/A2), *aph3/str*(A), *aph6/str*(B)	*mac*(A), *ma*c(B)	*tet*(A)		*cmr*		*qacEΔ1*	*sul1*, *sul2*	*dfrA1*, *dfrA8*
NTS01755	*bla*_LEN_, ***bla***_**SHV**_	*aad*(A1/A2), *aph3/str*(A), *aph6/str*(B)	*mac*(A), *ma*c(B)	*tet*(D)		*cmr*		*qacEΔ1*	*sul1*, *sul2*	*dfrA7*
NTS01793	***bla***_**SHV**_	*aph3/str*(A), *aph6/str*(B)								
NTS01936	*bla*_LEN_, ***bla***_**SHV**_									

bold indicates that microarray-detected *bla*_CTX-M_ or *bla*_SHV_ genes were PCR-confirmed (see S1 and S2 Tables). Results shown in parentheses indicates that *bla*_SHV_ was detected by PCR but not by microarray.

### Resistance to β-lactams

The ARDM v.2 content comprises probes for 52 β-lactamase genes, including 12 families of extended-spectrum β-lactamases (ESBLs) and 15 carbapenemases. The ARDM detected *bla*_SHV_, a chromosomal gene presumptively carried in all *K*. *pneumoniae* [23], in 63 isolates (70%), while PCR detected *bla*_SHV_ in an additional fourteen (S2 Table); 13 of the 90 isolates were negative for *bla*_SHV_ by both methods, but this may be due to point mutations within the primer regions (PCR) or regions used for hybridization on the microarray. β-lactamase inhibitors such as clavulanate and sulbactam are typically active against *Klebsiella* SHV-1 and TEM-1 lactamases, but one-third of the isolates tested here showed resistance to at least one of these inhibitors. While such resistance may arise from hyperproduction of β-SHV lactamases [24], this resistance was highly correlated to the presence of *bla*_TEM_ (*P*<0.0001), suggesting either TEM hyperproduction [25,26] or the possible presence of inhibitor-resistant TEM enzymes. The presence of *bla*_OXA-1-like_ genes–most often conferring resistance to clavulanate and sulbactam–can also potentially explain phenotypic inhibitor resistance in two strains (MHK01590, MHK05068), although *bla*_TEM_ genes are also present in both. However, strain MHK01305—positive for *bla*_OXA-1-like_, *bla*_TEM_, and *bla*_CTX-M-1_ family genes–is broadly susceptible to almost all tested β-lactams and lactam-inhibitor combinations, suggesting that either none of these genes are expressed or that the encoded gene products are non-functional.

Nine strains were resistant to at least one third or fourth generation cephalosporin (Table 1), with six classified as ESBL producers by the MicroScan. Five of the ESBL-producing isolates were positive for *bla*_CTX-M-1_-group genes (confirmed by PCR, see S1 and S2 Tables). An additional three isolates also carried *bla*_CTX-M-1_-family genes, two of which were resistant to the third and fourth generation cephalosporins tested but negative for ESBL production by Microscan; one of these (MHK04922) also carried *ampC*/*bla*_DHA_, which can mask the ESBL phenotype [27]. One isolate (NTS01708) was positive for the *bla*_CTX-M-2_-family, which was also confirmed by PCR. The *bla*_CTX-M-2_ amplicon sequence (NCBI Accession no. KX377894) identified this gene as encoding a protein most similar to CTX-M-2 (Toho 1), CTX-M-20, CTX-M-56, CTX-M-75, CTX-M-95, CTX-M-165, and KLUA-9. To our knowledge, this is the first time that a gene from the *bla*_CTX-M-2_-family has been identified within *Enterobacteriaceae* from East Africa. Interestingly, this *bla*_CTX-M-2_-positive isolate were susceptible to both of the lactam/inhibitor combinations tested and all other tested β-lactams except ampicillin, suggesting that this gene was not transcribed or that the encoded proteins was non-functional. None of the 90 isolates were positive for genes encoding the CTX-M-8 and CTX-M-9 families of ESBLs. The preferential carriage of CTX-M-1-type enzymes over other ESBLs agrees with other studies of this region [28,29].

Only three isolates were phenotypically resistant to either imipenem (one isolate) or meropenem (two isolates). However, none of the 15 carbapenemase genes represented on the ARDM v.2 were detected.

### Resistance to aminoglycosides

Isolates were tested for the presence of 44 different aminoglycoside resistance determinants. While only nine of the isolates were resistant to the three aminoglycosides tested, a relatively large number harbored genes commonly associated with aminoglycoside resistance: *aac(3)-III* (five isolates); *aac(6)-Ib* family (four isolates); *aadA1*/*A2* family (18 isolates); *aad*(A4) (one isolate); *aphA1* (seven isolates); *aph3/str*(A) (44 isolates); *aph6/str*(B) (47 isolates), and *rmtB* (one isolate). As the microarray cannot detect point mutations, we PCR-amplified and sequenced the *aac(6)-Ib* genes detected in four isolates to confirm that these alleles were not the *aac(6)-Ib-cr* variant conferring resistance to quinolones. The presence of *aac(3)-III* was correlated to phenotypic resistance to gentamicin and tobramycin (*P* < 0.0001) and *aac(6)-Ib* family genes to amikacin and tobramycin (*P* < 0.0001). Not surprisingly, the isolate harboring *rmtB*, which confers pan-resistance to aminoglycosides, was resistant to all three aminoglycosides.

### Resistance to tetracyclines, chloramphenicol

Almost half of the isolates were non-susceptible to tetracycline. Phenotypic resistance was positively correlated to the presence of a tetracycline resistance determinant (*P* < 0.0005), although 10 isolates harboring resistance genes were phenotypically sensitive. Of the 38 tetracycline resistance genes on the ARDM v.2, only four were detected: *tet*(A) (18%), *tet*(D) (12%), *tet*(B) (10%), and *tet*(G) (1%).

The ARDM v.2 chip also contains probes directed against 20 chloramphenicol resistance determinants. However, only four were detected in the tested population: *cmr* (32 isolates); two variants of *floR* originating from different species (one isolate); *cmlA* (one isolate); and *catA1/cat4* (seven isolates). Phenotypic resistance to chloramphenicol was not assessed.

### Resistance to quinolones

A single isolate (MHK02590) was resistant to both ciprofloxacin and levofloxacin, while the remainder were susceptible to one (three isolates) or both quinolones tested (86 isolates). The plasmid-mediated quinolone resistance gene, *qnrS*, was observed in two isolates, of which one displayed intermediate susceptibility for ciprofloxacin. None of the other plasmid-mediated quinolone resistance genes were detected (*norA*, *qnrA*, *qepA*, *aac(6)-Ib-cr*). The ARDM is unable to identify mutations in gyrase or helicase genes that confer high-level resistance to quinolones.

### Genes conferring resistance to macrolides, lincosamides, streptogramins, and ansamycins

Ansamycins and macrolides, lincosamide, and streptogramin (MLS) antibiotics are not typically considered clinically relevant for treatment of Gram-negative infections. However, some researchers have suggested that commensal Gram-negative organisms may serve as a reservoir of AMR genes that can be transferred to other pathogens and organisms responsible for severe intestinal infections [30,31,32,33]. For this reason, the ARDM v.2 chip content includes ten MLS resistance genes derived from Gram-negative species, in addition to 31 MLS resistance genes derived from Gram-positive species. As expected, none of the isolates tested were positive for any of the Gram-positive-derived MLS resistance determinants, but *Escherichia coli*-derived genes, *mph*(A)/*mph*(K), *mac*(A), and *mac*(B), were detected in six, 29, and 25 isolates, respectively. All isolates positive for *mac*(B) also harbored *mac*(A). PCR amplification and amplicon sequencing confirmed that the microarray-detected *mac*(A) and *mac*(B) sequences are analogous to those derived from *E*. *coli* (NCBI accession nos. KX377891 through KX377893), although Klebsiella-derived analogs were also detected. Analogous *mac*(A) and *mac*(B) genes derived from *Klebsiella* spp. are only 70% identical to the *E*. *coli* genes and can be discriminated from the *E*. *coli*-derived genes by hybridization to the ARDM and amplicon sequencing (S2 Table).

Two isolates were positive for the presence of the rifampicin resistance determinant, *arr*. The presence of *arr* and *mphA/mphK* within stool isolates of *K*. *pneumoniae*–while not clinically relevant in itself—may portend the spread of azithromycin or rifaximin resistance, respectively, to other intestinal pathogens, potentially limiting the effectiveness of these drugs for treatment of travelers' diarrhea [34,35].

### Resistance to sulfonamides, quaternary amines, streptothricin, and trimethoprim

Sixty percent of the tested isolates were resistant to SXT, a first line agent for treatment of enteric infections in many parts of Africa [33,36]. Phenotypic resistance to SXT was highly correlated to the presence of a sulfonamide or trimethoprim resistance determinant (*P* << 0.0001). Approximately half of the tested isolates harbored at least one of the 28 trimethoprim resistance genes present on the ARDM: *dfrA14* (18 isolates), *dfrA1* (nine isolates), *dfrA5* (seven isolates), *dfrA7* or *dfrA8* (5 isolates each), and *dfrA12*, *dfrA13/21/22/23* family, *dfrA15*, *dfrA16*, and *dfrA17* (three or fewer isolates each). The high rate of *dfrA14*-positive samples observed here contrasts with other studies showing a much higher proportion of *dfrA1* and *dfrA7* amongst African intestinal isolates [37,38]. Seven isolates harbored multiple *dfrA* genes.

Present in 52.2% of the tested isolates, *sul2* was the most frequently encountered sulfonamide resistance determinant. *Sul1* was detected in 28 isolates, 21 of which also harbored *sul2*. In agreement with other studies of the region [37,39], *sul3* was infrequently encountered (1 isolate).

Twenty-seven of the 28 isolates positive for *sul1* also harbored *qacEΔ*1. Although association of *qac* genes with phenotypic antiseptic resistance is currently under debate, co-carriage of *qacEΔ*1 with *sul1* within the 3'-conserved sequences of many class 1 integrons is often linked to the presence of other resistance genes, presumptively as gene cassettes within the integrons [40]. The presence of *intI1* –indicative of a class 1 integron—was confirmed in all *qacEΔ*1+/*sul1*+ isolates. *IntI1* was detected in 20 additional strains by PCR, indicating the absence of a full 3'-conserved sequence amongst almost half of the integrons detected here (S2 Table). Carriage of class 1 integrons with alternative structures has previously been documented within Kenya, albeit at lower rates [39]. Similarly, co-carriage of *dfrA1*, *aadA1/A2*, and *sat2* is often associated with the presence of class 2 integrons. PCR amplification of *intI2* confirmed the presence of class 2 integrons in the three isolates harboring all three genes.

## Discussion

With improvements in metagenomic sequencing and other methods to characterize intestinal microbiota, a number of recent studies have documented intestinal colonization with klebsiellae as a source of extra-intestinal infections [4] and an initial stage in many nosocomial infections [6,41]. Pertinent to the current study, intestinal klebsiellae and other *Enterobacteriaceae* may serve as reservoirs of AMR determinants, increasing the potential for highly resistant disease [10,12,42]. Here we have assessed a collection of 90 *Klebsiella* spp. intestinal isolates as a model for the accumulation and evolution of resistance assemblages within the gut of Kenyan individuals.

Our data suggest that there is some selective pressure for the establishment and maintenance of bacterial populations resistant to multiple antimicrobial compounds within this region. The high proportion of isolates that were classified as MDR (36.7%), in a sample population *not selected for resistance* underscores this point, although some bias may have resulted from recent antibiotic use by the participants (no participant medical histories were available for most samples). Specific to Kenya, widespread use of tetracycline in livestock production [43], use of SXT and chloramphenicol as first line therapeutics for typhoid [2,44], and prophylactic use of SXT in persons exposed to or infected with HIV [45] may have contributed to the high prevalence of resistance to these compounds. These results are in line with other studies in East Africa showing similar rates of resistance and carriage of AMR genes [46,47,48]. On the other hand, while ciprofloxacin and third generation cephalosporins are widely distributed in Kenya [49,50,51,52], their high costs limit their use [53,54,55,56]. Thus, it was not surprising that only a small percentage of the tested population was resistant to fluoroquinolones or third/fourth generation cephalosporins, with a correspondingly low number of isolates positive for genes conferring resistance to these compounds. Similarly, carbapenem resistance was observed in only three isolates, and none of the 15 carbapenemase genes on the ARDM v.2 were identified here, including those detected in previous studies of the region where higher carbapenem resistance was observed (e.g., *bla*_OXA-48_, *bla*_VIM_, *bla*_NDM_, *bla*_IMP_, *bla*_KPC_) [57,58,59]. Differences in the current dataset and those of other studies in East Africa may simply reflect the particular species studied (e.g., *E*. *coli*, *Klebsiella* spp.), age and medical histories of participants, or the sample sources (e.g., urine, blood, stool). Alternatively, our results may suggest that availability and use of carbapenems are lower in Kenya than elsewhere in the region [60].

A large number of *K*. *pneumoniae* strains hybridized to the *mac*(A) and *mac*(B) probes derived from *E*. *coli* genes, although isolates carrying variants from both species were also identified (S2 Table). Interestingly, the presence of *E*. *coli*-derived *mac*(A)/*mac*(B) genes was also correlated with the presence of sequences hybridizing to an *E*. *coli*-derived *cmr* gene (*P* <0.0001), which is only ~80% identical to the *Klebsiella* spp. homolog. BLAST searches of the *E*. *coli*-derived *mac*(AB) sequences indicated that these sequences have not previously been documented in any klebsiellae.

The breadth of genes on the microarray allowed us to detect multiple classes of resistance determinants, which may suggest the presence of integrons and/or plasmids associated with AMR. Strain MHK02590, isolated at Mbagathi District Hospital in Nairobi, was resistant to all tested antimicrobials except carbapenems and harbored 21 resistance determinants. Interestingly, Kariuki and colleagues [61] recently isolated an IncHI2 plasmid, pKST313, from a Kenyan *Salmonella typhimurium* carrying 11 of these determinants. While we did not attempt to confirm the presence of pKST313 in strain MHK02590, isolation of this strain within the Nairobi metropolitan area where pKST313 was first identified suggests that this plasmid may be circulating within this urban setting.

This study had several limitations. As with any molecular method, genotype is not always fully predictive of phenotype. Though statistically valid genotypic/phenotypic correlations could be made for many genes in this study, a disconnect was observed between the presence of several β-lactamase and dihydrofolate reductase genes and the predicted resistance profiles. These discrepancies could be due to poor gene expression, non-functionality of the expressed gene products, or the presence of other genes or mechanisms not addressed. On the other hand, we were unable to identify the molecular mechanisms for carbapenem or fluoroquinolone non-susceptibility observed in a number of samples. While carbapenem resistance was likely due to the presence of a carbapenemase gene not currently included in the ARDM chip content, fluoroquinolone resistance is likely due to mutations in DNA gyrase and topoisomerase genes, *gyrA* and *parC* [62,63]. The ARDM cannot detect these mutations. In such an instance, a more comprehensive technique such as whole genome sequencing (WGS) might provide the needed information. An additional advantage of WGS is the ability to discriminate closely related alleles and identification of changes in regulatory sequences affecting gene expression. However, WGS may also miss the presence of important genes or point mutations if coverage is insufficient or error rates are too high [64]. Nonetheless, molecular approaches such as microarray hybridization and WGS can assist in tracking the epidemiological development and spread of AMR, a benefit not realized through phenotypic testing.

Despite these limitations, we identified a high prevalence of MDR amongst a collection of Kenyan *Klebsiella* spp. stool isolates not specifically selected for their resistance characteristics. In most cases, phenotypic resistance was highly correlated to the presence of appropriate AMR determinants. While our results suggest that selective pressure exists for carriage of genes conferring resistance to tetracyclines, phenicols, trimethoprim, and sulfonamides, resistance to fluoroquinolones, third- and fourth-generation cephalosporins, and carbapenems was observed in only a small number of isolates, likely commensurate with regional usage. The wide variety of resistance determinants detected, the large number of isolates harboring five or more of these genes (65.5%) and the high prevalence of MDR phenotype (36.7%) underscore the need for more effective, targeted public health policies and infection control/prevention measures than those likely implemented in the population tested. Timely public health intervention to new and emerging sources of resistance are always important–and unfortunately often not available—in developing countries where access to second- and third-line antimicrobials may be limited.

## Supporting information

S1 Table PCR primers used for confirmation of specific AMR and integrase genes. (DOCX)

S2 Table Comparison of resistance genes detected thru microarray hybridization and by PCR. (DOCX)

## References

[1] World Health Organization, http://www.who.int/drugresistance/documents/surveillancereport/en/; 27 May 2015.

[2] Global Antibiotic Resistance Partnership-Kenya Working Group. Situational analysis and recommendations—Antibiotic use and resistance in Kenya. Washington, DC Center for Disease Dynamics, Economics & Policy 2011.

[3] Thi PLN, Yassibanda S, Aidara A, Le Bouguénec C, Germani Y. Enteropathogenic Klebsiella pneumoniae HIV-Infected Adults, Africa. Emerg Infect Dis. 2003;9: 135–137. 10.3201/eid0901.020138 PMC2873755 12533299

[4] Fung C-P, Lin Y-T, Lin J-C, Chen T-L, Yeh K-M, Chang R-Y, et al *Klebsiella pneumoniae* in gastrointestinal tract and pyogenic liver abscess Emerg Infect Dis. 2012;18: 1322–1325. doi: 10.3201/eid1808.111053 22840473 10.3201/eid1808.111053 PMC3414011

[5] Selden R, Lee S, Wang W, Bennett JV, Eickhoff TC. Nosocomial klebsiella infections: intestinal colonization as a reservoir. Ann Intern Med. 1971;74: 657–664. 5559431 10.7326/0003-4819-74-5-657

[6] Martin RM, Cao J, Brisse S, Passet V, Wu W, Zhao L, et al Molecular epidemiology of colonizing and infecting isolates of *Klebsiella pneumoniae*. mSphere. 2016;1: e00261–00216. doi: 10.1128/mSphere.00261-16 27777984 10.1128/mSphere.00261-16 PMC5071533

[7] Wanyiri JW, Kanyi H, Maina S, Wang DE, Ngugi P, O'Connor R, et al Infectious diarrhoea in antiretroviral therapy-naïve HIV/AIDS patients in Kenya. Trans R Soc Trop Med Hyg. 2013;107: 631–638. doi: 10.1093/trstmh/trt078 24026463 10.1093/trstmh/trt078 PMC3840903

[8] Huddleston JR. Horizontal gene transfer in the human gastrointestinal tract: potential spread of antibiotic resistance genes. Infect Drug Resist. 2014;7: 167–176. doi: 10.2147/IDR.S48820 25018641 10.2147/IDR.S48820 PMC4073975

[9] Schjørring S, Krogfelt KA. Assessment of bacterial antibiotic resistance transfer in the gut. Int J Microbiol. 2011;2011: article 312956. 10.1155/2011/312956 PMC3034945 21318188

[10] Salyers AA, Gupta A, Wang Y. Human intestinal bacteria as reservoirs for antibiotic resistance genes. Trends Microbiol. 2004;12: 412–416. doi: 10.1016/j.tim.2004.07.004 15337162 10.1016/j.tim.2004.07.004

[11] Podschun R, Ullmann U. *Klebsiella* spp. as nosocomial pathogens: epidemiology, taxonomy, typing methods, and pathogenicity factors. Clin Microbiol Rev. 1998;11: 589–603. 9767057 10.1128/cmr.11.4.589 PMC88898

[12] Schjorring S, Struve C, Krogfelt KA. Transfer of antimicrobial resistance plasmids from *Klebsiella pneumoniae* to *Escherichia coli* in the mouse intestine. J Antimicrob Chemother. 2008;62: 1086–1093. doi: 10.1093/jac/dkn323 18703526 10.1093/jac/dkn323 PMC2566516

[13] Sidjabat HE, Silveira FP, Potoski BA, Abu-Elmagd KM, Adams-Haduch JM, Paterson DL, et al Interspecies spread of *Klebsiella pneumoniae* carbapenemase gene in a single patient. Clin Infect Dis. 2009;49: 1736–1738. doi: 10.1086/648077 19886795 10.1086/648077

[14] Gona F, Barbera F, Pasquariello AC, Grossi P, Gridelli B, Mezzatesta ML, et al *In vivo* multiclonal transfer of *bla*_KPC-3_ from *Klebsiella pneumoniae* to *Escherichia coli* in surgery patients. Clin Microbiol Infect. 2014;20: O633–O635. doi: 10.1111/1469-0691.12577 24476498 10.1111/1469-0691.12577

[15] Goren MG, Carmeli Y, Schwaber JM, Chmelnitsky I, Schechner V, Navon-Venezia S. Transfer of carbapenem-resistant plasmid from *Klebsiella pneumoniae* ST258 to *Escherichia coli* in patient. Emerg Infect Dis. 2010;16: 1014–1017. doi: 10.3201/eid1606.091671 20507761 10.3201/eid1606.091671 PMC3086234

[16] Guan J, Liu S, Lin Z, Li W, Liu X, Chen D. Severe sepsis facilitates intestinal colonization by extended-spectrum-β-lactamase-producing *Klebsiella pneumoniae* and transfer of the SHV-18 resistance gene to *Escherichia coli* during antimicrobial treatment. Antimicrob Agents Chemother. 2014;58: 1039–1046. doi: 10.1128/AAC.01632-13 24277046 10.1128/AAC.01632-13 PMC3910833

[17] Pavlinac PB, John-Stewart GC, Naulikha JM, Onchiri FM, Denno DM, Odundo EA, et al High-risk enteric pathogens associated with HIV-infection and HIV-exposure in Kenyan children with acute diarrhea. AIDS. 2014;28: 2287–2296. doi: 10.1097/QAD.0000000000000396 25028987 10.1097/QAD.0000000000000396 PMC4346243

[18] CLSI. Performance standards for antimicrobial susceptbility testing; twenty-fifth informational supplement. Wayne, PA: Clinical and Laboratory Standards Institute 2015.

[19] Taitt CR, Leski TA, Stockelman M, Craft DW, Zurawski DV, Kirkup BC Jr, et al Antimicrobial resistance determinants in *Acinetobacter baumannii* isolates taken from military treatment facilities. Antimicrob Agents Chemother. 2014;58: 767–781. doi: 10.1128/AAC.01897-13 24247131 10.1128/AAC.01897-13 PMC3910874

[20] Taitt CR, Leski TA, Heang V, Ford GW, Prouty MG, Newell SW, et al Antimicrobial resistance genotypes and phenotypes from multidrug-resistant bacterial wound infection isolates in Cambodia. J Glob Antimicrob Resist. 2015;3: 198–204. doi: 10.1016/j.jgar.2015.05.006 27873709 10.1016/j.jgar.2015.05.006

[21] Leski TA, Vora GJ, Barrows BR, Pimentel G, House BL, Nicklasson M, et al Molecular characterization of multidrug resistant hospital isolates using the Antimicrobial Resistance Determinant Microarray. PLOS ONE. 2013;8: e69507 doi: 10.1371/journal.pone.0069507 23936031 10.1371/journal.pone.0069507 PMC3723915

[22] Magiorakos AP, Srinivasan A, Carey RB, Carmeli Y, Falagas ME, Giske CG, et al Multidrug-resistant, extensively drug-resistant and pandrug-resistant bacteria: an international expert proposal for interim standard definitions for acquired resistance. Clin Microbiol Infect. 2012;18: 268–281. doi: 10.1111/j.1469-0691.2011.03570.x 21793988 10.1111/j.1469-0691.2011.03570.x

[23] Ford PJ, Avison MB. Evolutionary mapping of the SHV b-lactamase and evidence for two separate IS*26*-dependent *bla*_SHV_ mobilization events from the *Klebsiella pneumoniae* chromosome. J Antimicrob Chemother. 2004;54: 69–75. doi: 10.1093/jac/dkh251 15163647 10.1093/jac/dkh251

[24] French GL, Shannon KP, Simmons N. Hospital outbreak of *Klebsiella pneumoniae* resistant to broad-spectrum cephalosporins and b-lactam-b-lactamase inhibitor combinations by hyperproduction of SHV-5 b-lactamase. J Clin Microbiol. 1996;34: 358–363. 8789016 10.1128/jcm.34.2.358-363.1996 PMC228798

[25] Shannon K, Williams H, King A, Philipps I. Hyperproduction of TEM-1 β-lactamase in clinical isolates of *Escherichia coli* serotype O15. FEMS Microbiol Lett. 1990;67: 319–324. 10.1016/0378-1097(90)90016-j 2182386

[26] Wu PJ, Shannon K, Phillips I. Mechanisms of hyperproduction of TEM-1 β-lactamase by clinical isolates of *Escherichia coli*. J Antimicrob Chemother. 1995;36: 927–939. 8821592 10.1093/jac/36.6.927

[27] Garrec H, Drieux-Rouzet L, Golmard JL, Jarlier V, Robert J. Comparison of nine phenotypic methods for detection of extended-spectrum β-lactamase production by Enterobacteriaceae. J Clin Microbiol. 2011;49: 1048–1057. doi: 10.1128/JCM.02130-10 21248086 10.1128/JCM.02130-10 PMC3067698

[28] Albrechtova K, Dolejska M, Cizek A, Tausova D, Klimes J, Bebora L, et al Dogs of nomadic pastoralists in Northern Kenya are reservoirs of plasmid-mediated cepahlosporin- and quinolone-resistant *Escherichi coli*, including pandemic clone B2-O25-ST131. Antimicrob Agents Chemother. 2012;56: 4013–4017. doi: 10.1128/AAC.05859-11 22508313 10.1128/AAC.05859-11 PMC3393422

[29] Kiiru J, Kariuki S, Goddeeris B, Buraye P. Analysis of b-lactamase phenotypes and carriage of selected b-lactamase genes among *Escherichia coli* strains obtained from Kenyan patients during an 18-year period. BMC Microbiol. 2012;12: 155 doi: 10.1186/1471-2180-12-155 22838634 10.1186/1471-2180-12-155 PMC3464591

[30] Nguyen MCP, Woerther P-L, Bouvet M, Andremont A, Leclercq R, Canu A. *Escherichia coli* as reservoir for macrolide resistance genes. Emerg Infect Dis. 2009;15: 1648–1650. doi: 10.3201/eid1510.090696 19861064 10.3201/eid1510.090696 PMC2866414

[31] Kariuki S. Antimicrobial resistance in enteric pathogens in developing countries In: Sosa AdJ, Byarugaba DK, Amabile-Cuevas CF, Hsueh PR, Kariuki S et al, editors. Antimicrobial Resistance in Developing Countries. New York: Springer; 2010. pp.

[32] Nys S, Okeke IN, Kariuki S, Dinant GJ, Driessen C, Stobberingh EE. Antibiotic resistance of faecal *Escherichia coli* from healthy volunteers from eight developing countries. J Antimicrob Chemother. 2004;54: 952–955. doi: 10.1093/jac/dkh448 15471998 10.1093/jac/dkh448

[33] Okeke IN, Aboderin OA, Byarugaba DK, Ojo KK, Opintan JA. Growing problem of multidrug-resistant enteric pathogens in Africa. Emerg Infect Dis. 2007;13: 1640–1646. doi: 10.3201/eid1311.070674 18217545 10.3201/eid1311.070674 PMC3375797

[34] DuPont HL. For the record: A history of the definition & management of travelers' diarrhea In: Brunette GW, Kozarsky PE, Gershman MD, Magill AJ, Ostroff SM et al, editors. CDC Health Information for International Travel. Atlanta, GA: CDC; 2016. pp.

[35] Steffen R, Sack DA, Riopel L, Jiang ZD, Sturchler M, Ericsoon CD, et al Therapy of travelers' diarrhea with rifaximin on various continents. Am J Gastroenterol. 2003;98: 1073–1078. doi: 10.1111/j.1572-0241.2003.07283.x 12809830 10.1111/j.1572-0241.2003.07283.x

[36] Vila J, Vargas M, Casals C, Urassa H, Mshinda H, Schellemberg D, et al Antimicrobial resistance of diarrheagenic Escherichia coli isolated from children under the age of 5 years from Ifakara, Tanzania. Antimicrob Agents Chemother. 1999;43: 3022–3024. 10582903 10.1128/aac.43.12.3022 PMC89608

[37] Frank T, Gautier V, Talarmin A, Bercion R, Arlet G. Characterization of sulphonamide resistance genes and class 1 integron gene cassettes in Enterobacteriaceae, Central African Republic (CAR). J Antimicrob Chemother. 2007;59: 742–745. doi: 10.1093/jac/dkl538 17350987 10.1093/jac/dkl538

[38] Labar AS, Millman JS, Ruebush E, Opintan JA, Bishar RA, Aboderin AO, et al Regional dissemination of a trimethoprim-resistance gene cassette via a successful transposable element. PLOS ONE. 2012;7: e38142 doi: 10.1371/journal.pone.0038142 22666464 10.1371/journal.pone.0038142 PMC3364232

[39] Kiiru J, Butaye P, Goddeeris B, Kariuki S. Analysis for prevalence and physical linkages amongst integrons, ISEcp1, ISCR1, Tn21 and Tn7 encountered in *Escherichia coli* strains from hospitalized and non-hospitalized patients in Kenya during a 19-year period (1992–2011). BMC Microbiol. 2013;13: 109 doi: 10.1186/1471-2180-13-109 23682924 10.1186/1471-2180-13-109 PMC3663672

[40] Jaglic Z, Cervinkova D. Genetic basis of resistance to quaternary ammonium compounds—the *qac* genes and their role: a review. Vet Med (Praha). 2012;57: 275–281.

[41] Conlan S, Park M, Deming C, Thomas PJ, Young AC, Coleman H, et al Plasmid dynamics in KPC-positive *Klebsiella pneumoniae* during long-term patient colonization. mBio. 2016;7: e00742–00716. doi: 10.1128/mBio.00742-16 27353756 10.1128/mBio.00742-16 PMC4937214

[42] van Schaik W. The human gut resistome. Philos Trans R Soc London, Ser B. 2015;370: 20140087. 10.1098/rstb.2014.0087 PMC4424436 25918444

[43] Mitema ES, Kikuvi GM, Wegener HC, Stohr K. An assessment of antimicrobial consumption in food producing animals in Kenya. J Vet Pharmacol Ther. 2001;24: 385–390. 11903868 10.1046/j.1365-2885.2001.00360.x

[44] Ministry of Medical Services and Ministry of Public Health and Sanitation. Kenya essential medicines list. Nairobi: Government of Kenya with the World Health Organization 2010.

[45] Bwakura-Dangarembizi M, Kendall L, Bakeera-Kitaka S, Nahirya-Ntege P, Keishanyu R, Nathoo K, et al A randomized trial of prolonged co-trimoxazole in HIV-infected children in Africa. N Engl J Med. 2014;370: 41–53. doi: 10.1056/NEJMoa1214901 24382064 10.1056/NEJMoa1214901 PMC4264559

[46] Hamel MJ, Greene C, Chiller T, Ouma P, Polyak C, Otieno K, et al Does cotrimoxazole prophylaxis for the prevention of HIV-associated opportunistic infections select for resistant pathogens in Kenyan adults? Am J Trop Med Hyg. 2008;79: 320–330. 18784222

[47] Nelson N, Joshi M, Kirika R. Antimicrobial resistance: The need for action in the East, Central, and Southern Africa region. Arlington, VA: USAID 2009.

[48] Mwambete KD, Kamuhabwa AAR. Resistance of commensal intestinal *Escherichia coli* and other enterics to co-trimoxazole and commonly used antibiotics in HIV/AIDS patients. Clin Microbiol Rev. 2013;3: 1000134.

[49] Kohli R, Omuse G, Revathi G. Antibacterial susceptibility patterns of blood stram isolates in patients investigated at the Aga Khan University Hospital, Nairobi. East Afr Med J. 2010;87: 74–80. 23057259 10.4314/eamj.v87i2.60592

[50] Blomberg S, Jureen R, Manji KP, Tamim BS, Mwakagile DS, Urassa WK, et al High rate of fatal cases of pediatric septicemia caused by Gram-negative bacteria with extended-spectrum β-lactamases in Dar es Salaam, Tanzania. J Clin Microbiol. 2005;43. 10.1128/JCM.43.2.745-749.2005 PMC548071 15695674

[51] Maina D, Revathi G, Kariuki S, Ozwara H. Genotypes and cephalosporin susceptibility in extended-spectrum beta-lactamase producing Enterobacteriacea in the community. J Infect Dev Ctries. 2012;6: 470–477. 22706188 10.3855/jidc.1456

[52] Muvunyi CM, Masaisa F, Bayingana C, Mutesa L, Musemakweri A, Muhirwa G, et al Decreased susceptibility to commonly used antimicrobial agents in bacterial pathogens isolated from urinary tract infections in Rwanda: Need for new antimicrobial guidelines. Am J Trop Med Hyg. 2011;84: 923–928. doi: 10.4269/ajtmh.2011.11-0057 21633029 10.4269/ajtmh.2011.11-0057 PMC3110351

[53] Rogawski ET, Platts-Mills JA, Seidman JC, John S, Mahfuz M, Ulak M, et al Use of antibiotics in children younger than two years in eight countries: a prospective cohort study. Bull World Health Organ. 2017;95: 49–61. doi: 10.2471/BLT.16.176123 28053364 10.2471/BLT.16.176123 PMC5180352

[54] Cameron A, Ewen M, Ross-Degnan D, Ball D, Laing R. Medicine prices, availability, and affordability in 36 developing and middle-income countries: a secondary analysis. The Lancet. 2009;373: 240–249. 10.1016/S0140-6736(08)61762-6 19042012

[55] Ministry of Medical Services and Ministry of Public Health and Sanitation. Kenya: Service provision assessment survey 2010. Nairobi, Kenya: Ministry of Medical Services and Ministry of Public Health and Sanitation 2010.

[56] World Health Organization. Medicine prices in Kenya. Geneva: World Health Organization 2004.

[57] Okoche D, Asiimwe BB, Katabazi FA, Kato L, Najjuka CF. Prevalence and characterization of carbapenem-resistant Enterobacteriaceae isolated from Mulago National Referral Hospital, Uganda. PLOS ONE. 2015;10: e0135745 doi: 10.1371/journal.pone.0135745 26284519 10.1371/journal.pone.0135745 PMC4540283

[58] Mushi MF, Mshana SE, Imirzalioglu C, Bwanga F. Carbapenemase genes among multidrug resistant Gram negative clinical isolates from a tertiary hospital in Mwanza, Tanzania. Biomed Res Int. 2014;2014: 303104 doi: 10.1155/2014/303104 24707481 10.1155/2014/303104 PMC3953670

[59] Poirel L, Revathi G, Bernabeu S, Nordmann P. Detection of NDM-1-producing *Klebsiella pneumoniae* in Kenya. Antimicrob Agents Chemother. 2011;55: 934–936. doi: 10.1128/AAC.01247-10 21115785 10.1128/AAC.01247-10 PMC3028766

[60] Musembi KP (2016) Drug consumption patterns with clinical and financial implications at Kenyatta National Hospital. Nairobi, Kenya: University of Nairobi.

[61] Kariuki S, Okoro C, Kiiru J, Njoroge S, Omuse G, Langridge G, et al Ceftriaxone-resistant *Salmonella enterica* serotype *Typhimurium* sequence type 313 from Kenyan patients is associated with the *bla*_CTX-M-15_ gene on a novel IncHI2 plasmid. Antimicrob Agents Chemother. 2015;59: 3133–3139. doi: 10.1128/AAC.00078-15 25779570 10.1128/AAC.00078-15 PMC4432211

[62] Ruiz J. Mechanisms of resistance to quinolones: target alterations, decreased accumulation, and DNA gyrase protection. J Antimicrob Chemother. 2003;51: 1109–1117. doi: 10.1093/jac/dkg222 12697644 10.1093/jac/dkg222

[63] Weigel LM, Steward CD, Tenover FC. *gyrA* mutations associated with fluoroquinolone resistance in eight species of *Enterobacteriaceae*. Antimicrob Agents Chemother. 1998;42: 2661–2667. 9756773 10.1128/aac.42.10.2661 PMC105915

[64] Sims D, Sudbery I, Ilott NE, Heger A, Ponting CP. Sequencing depth and coverage: key considerations in genomic analyses. Nat Rev Genet. 2014;15: 121–132. doi: 10.1038/nrg3642 24434847 10.1038/nrg3642

