# Supplementary material for: Antimicrobial resistance of Klebsiella pneumoniae stool isolates circulating in Kenya
Source: PLoS One. 2017 Jun 2;12(6):e0178880. doi: 10.1371/journal.pone.0178880 (PMC5456380; doi:10.1371/journal.pone.0178880)
Supplement: S1 Table — (DOCX) [file pone.0178880.s001.docx]

S1 Table: PCR primers used for confirmatory testing

| **Primer name** | **Sequence** | **Target Gene** | **Source** |
| --- | --- | --- | --- |
| Int1F | CAG TGG ACA TAA GCC TGT TC | *intI1* | [[1](#_ENREF_1)] |
| Int1R | CCC GAG GCA TAG ACT GTA |  |  |
| Int2F | TTG CGA GTA TCC ATA ACC TG | *intI2* | [[2](#_ENREF_2)] |
| Int2R | TTA CCT GCA CTG GAT TAA GC |  |  |
| CTX-M-F | ATG TGC AGY ACC AGT AAR GTK ATG GC | *bla*_CTX-M_ (universal primers) | [[3](#_ENREF_3)] |
| CTX-M-R | TGG GTR AAR TAR GTS ACC AGA AYC AGC GG |  |  |
| SHV-F | CTT TAT CGG CCC TCA CTC AA | *bla*_SHV_ (universal primers) |  |
| SHV-R | AGG TGC TCA TCA TGG GAA AG |  |  |
| aac(6’)-Ib-F | TTGCGATGCTCTATGAGTGGCTA | *aac*(6’)-Ib and *aac*(6’)-Ib-cr | [4] |
| aac(6’)-Ib-R | CTCGAATGCCTGGCGTGTTT |  |  |
| macAB-F(EC) | TTT CCG CTC AAG ACC AGC AT | *mac*(A)-*mac*(B) (*E. coli*) | this work |
| macAB-R(EC) | GAT CCC TCT GTC GGC GTT AG |  |  |
| macA-F(KP) | CTG AAA ACG CTG CAC GTC AA | *mac*(A) (*K. pneumoniae*) | this work |
| macA-R(KP) | GCA CGG TAA ACC ACG CTT TT |  |  |

1. Koeleman JG, Stoof J, Van Der Bijl MW, Vandenbroucke-Grauls CM, Savelkoul PH. Identification of epidemic strains of *Acinetobacter baumannii* by integrase gene PCR. J Clin Microbiol 2000;39: 8.

2. Ploy MC, Denis F, Courvalin P, Lambert J. Molecular characterization of integrons in *Acinetobacter baumannii*: description of a hybrid class 2 integron. Antimicrob Agents Chemother 2000;44: 2684.

3. Fang H, Ataker F, Hedin G, Dornbusch K. Molecular epidemiology of extended-spectrum β-lactamases among Escherichia coli isolates collected in a Swedish hospital and its associated health care facilities from 2001 to 2006. J Clin Microbiol 2008;46: 707.

4. Park CH, Robicsek A, Jacoby GA, Sahm D, Hooper DC. Prevalence in the United States of *aac(6')-Ib-cr* encoding a coprofloxacin-modifying enzyme. Antimicrob Agents Chemother 2006;50: 3953-3955
