# Supplementary material for: Antimicrobial resistance of Klebsiella pneumoniae stool isolates circulating in Kenya
Source: PLoS One. 2017 Jun 2;12(6):e0178880. doi: 10.1371/journal.pone.0178880 (PMC5456380; doi:10.1371/journal.pone.0178880)
Supplement: S2 Table — (DOCX) [file pone.0178880.s002.docx]

S2 Table. Comparison of microarray hybridization results with confirmatory PCRs^a^

| Isolate no. | *bla*_SHV_ | | *bla*_CTX-M_ families | | | *mac*(A), *mac*(B) | | | | Class 1 integrons | | | Class 2 integrons | | | |
| --- | --- | --- | --- | --- | --- | --- | --- | --- | --- | --- | --- | --- | --- | --- | --- | --- |
|  | ARDM | PCR | ARDM | | PCR | *ARDM* | | *PCR* | | *ARDM* | | *PCR* | *ARDM* | | | *PCR* |
|  |  |  | *bla*_CTX-M-1_ | *bla*_CTX-M-2_ | universal *bla*_CTX-M_ | *mac*(A) | *mac*(B) | *E coli*-specific^b^ | *Klebsiella-*specific | *qacEΔ1* | *sul1* | *intI1* PCR | *sat2* | *aad*(A1/A2) family | *dfrA1* | *intI2* |
| MHK00504 | pos^c^ | pos | neg | neg |  | pos | pos | pos | pos | neg | neg | neg | pos | pos | pos | pos |
| MHK01305 | neg | pos | pos | neg | pos | pos | pos | pos | pos | pos | pos | pos | neg | neg | neg |  |
| MHK01419 | neg | pos | neg | neg |  | pos | neg | pos | pos | neg | neg | pos | neg | neg | neg |  |
| MHK01814 | pos | neg | neg | neg |  | pos | pos | pos | pos | pos | pos | pos | neg | neg | neg |  |
| MHK02123 | neg | neg | neg | neg |  | neg | neg |  |  | neg | neg | pos | neg | neg | neg |  |
| MHK02126 | neg | neg | neg | neg |  | neg | neg |  |  | neg | neg | neg | neg | neg | neg |  |
| MHK02178 | neg | neg | neg | neg |  | pos | pos | pos | pos | neg | neg | pos | pos | pos | neg | pos |
| MHK02303 | pos | pos | neg | neg |  | pos | pos | pos | pos | neg | neg | pos | neg | neg | neg |  |
| MHK02499 | neg | neg | pos | neg | pos | pos | pos | pos | neg | neg | neg | pos | neg | neg | neg |  |
| MHK02590 | pos | neg | pos | neg | pos | pos | pos | pos | neg | neg | neg | neg | neg | pos | neg |  |
| MHK02631 | pos | pos | neg | neg |  | pos | pos | pos | pos | neg | neg | pos | neg | neg | neg |  |
| MHK02678 | neg | pos | neg | neg |  | pos | pos | pos | pos | neg | neg | neg | neg | neg | neg |  |
| MHK02690 | neg | pos | neg | neg |  | pos | pos | pos | pos | pos | pos | pos | pos | pos | pos | pos |
| MHK02780 | pos | pos | neg | neg |  | pos | pos | pos | pos | neg | neg | pos | neg | neg | neg |  |
| MHK03026 | pos | pos | neg | neg |  | neg | neg |  |  | neg | neg | pos | neg | neg | neg |  |
| MHK04212 | neg | pos | neg | neg |  | neg | neg |  |  | neg | neg | pos | neg | neg | neg |  |
| MHK04617 | pos | pos | neg | neg |  | neg | neg |  |  | pos | pos | pos | neg | neg | neg |  |
| MHK04622 | pos | pos | neg | neg |  | neg | neg |  |  | neg | neg | neg | neg | neg | neg |  |
| MHK04775 | pos | pos | neg | neg |  | neg | neg |  |  | neg | neg | neg | neg | neg | neg |  |
| MHK04776 | pos | pos | neg | neg |  | neg | neg |  |  | pos | pos | pos | neg | neg | neg |  |
| MHK04777 | pos | neg | neg | neg |  | neg | neg | neg |  | pos | pos | pos | neg | neg | neg |  |
| MHK04779 | pos | pos | neg | neg |  | neg | neg |  |  | neg | neg | neg | neg | neg | neg |  |
| MHK04786 | neg | pos | neg | neg |  | neg | neg | neg |  | neg | neg | pos | neg | neg | neg |  |
| MHK04792 | pos | pos | neg | neg |  | neg | neg | neg |  | neg | neg | neg | neg | neg | neg |  |
| MHK04804 | pos | neg | neg | neg |  | neg | neg |  |  | pos | pos | pos | neg | neg | neg |  |
| MHK04812 | neg | pos | neg | neg |  | pos | pos | pos | pos | pos | pos | pos | neg | pos | neg |  |
| MHK04813 | neg | pos | neg | neg |  | pos | pos | pos | pos | neg | neg | pos | neg | neg | neg |  |
| MHK04819 | pos | pos | neg | neg |  | neg | neg |  |  | pos | neg | pos | neg | pos | neg |  |
| MHK04821 | neg | pos | neg | neg |  | neg | neg |  |  | pos | pos | pos | neg | neg | neg |  |
| MHK04822 | pos | pos | neg | neg |  | neg | neg | neg |  | neg | pos | pos | neg | neg | neg |  |
| MHK04834 | pos | pos | neg | neg |  | neg | neg |  |  | neg | neg | neg | neg | neg | neg |  |
| MHK04838 | pos | pos | neg | neg |  | neg | neg |  |  | neg | neg | neg | neg | neg | neg |  |
| MHK04847 | pos | pos | neg | neg |  | neg | neg | neg |  | neg | neg | neg | neg | neg | neg |  |
| MHK04864 | neg | pos | neg | neg |  | neg | neg |  |  | neg | neg | neg | neg | neg | neg |  |
| MHK04872 | pos | neg | neg | neg |  | neg | neg |  |  | pos | pos | pos | neg | neg | neg |  |
| MHK04885 | pos | pos | neg | neg |  | neg | neg | neg |  | pos | pos | pos | neg | pos | pos |  |
| MHK04900 | pos | pos | neg | neg |  | pos | pos | pos | pos | neg | neg | neg | neg | neg | neg |  |
| MHK04904 | pos | pos | neg | neg |  | neg | neg |  |  | neg | neg |  | neg | neg | neg |  |
| MHK04908 | pos | pos | neg | neg |  | neg | neg | neg |  | pos | pos | pos | neg | neg | neg |  |
| MHK04919 | pos | neg | neg | neg |  | pos | pos | pos | pos | neg | neg | pos | neg | neg | neg |  |
| MHK04922 | pos | pos | pos | neg | pos | neg | neg |  |  | pos | pos | pos | neg | neg | neg |  |
| MHK04923 | pos | pos | neg | neg |  | neg | neg |  |  | neg | neg | neg | neg | neg | neg |  |
| MHK04926 | pos | pos | neg | neg |  | neg | neg |  |  | neg | neg | neg | neg | neg | neg |  |
| MHK04928 | pos | pos | neg | neg |  | neg | neg |  |  | neg | neg | neg | neg | neg | neg |  |
| MHK04930 | pos | neg | pos | neg | pos | neg | neg |  |  | pos | pos | pos | neg | pos | neg |  |
| MHK04941 | pos | pos | neg | neg |  | neg | neg |  |  | neg | neg | neg | neg | neg | neg |  |
| MHK04943 | pos | pos | neg | neg |  | neg | neg | neg |  | neg | neg | neg | neg | neg | neg |  |
| MHK04946 | neg | neg | neg | neg |  | neg | neg |  |  | neg | neg | neg | neg | neg | neg |  |
| MHK04947 | neg | pos | neg | neg |  | pos | neg | pos | pos | neg | neg | pos | neg | neg | neg |  |
| MHK04948 | pos | pos | neg | neg |  | neg | neg |  |  | neg | neg | neg | neg | neg | neg |  |
| MHK04957 | pos | pos | neg | neg |  | neg | neg |  |  | neg | neg | neg | neg | neg | neg |  |
| MHK04960 | pos | pos | neg | neg |  | neg | neg |  |  | neg | neg | neg | neg | neg | neg |  |
| MHK04967 | pos | neg | neg | neg |  | neg | neg |  |  | pos | pos | pos | neg | neg | neg |  |
| MHK04980 | pos | pos | neg | neg |  | neg | neg | neg |  | neg | neg |  | neg | neg | neg |  |
| MHK04983 | neg | neg | neg | neg |  | neg | neg |  |  | pos | pos | pos | neg | pos | neg |  |
| MHK04984 | pos | pos | neg | neg |  | neg | neg |  |  | neg | neg | neg | neg | neg | neg |  |
| MHK05010 | pos | pos | neg | neg |  | pos | pos | pos | pos | neg | neg | pos | neg | neg | neg |  |
| MHK05013a | neg | neg | neg | neg | neg | neg | neg |  |  | pos | pos | pos | neg | pos | neg |  |
| MHK05013b | pos | pos | neg | neg |  | neg | neg |  |  | neg | neg | pos | neg | neg | neg |  |
| MHK05014a | neg | neg | neg | neg |  | neg | neg |  |  | neg | neg | neg | neg | neg | neg |  |
| MHK05014b | neg | neg | neg | neg |  | neg | neg |  |  | neg | neg | neg | neg | neg | neg |  |
| MHK05017 | pos | pos | neg | neg |  | neg | neg |  |  | neg | neg | neg | neg | neg | neg |  |
| MHK05018 | neg | neg | neg | neg |  | neg | neg |  |  | neg | neg | pos | neg | neg | neg |  |
| MHK05018-1b | pos | neg | neg | neg |  | pos | pos | pos | pos | neg | neg | neg | neg | neg | neg |  |
| MHK05021 | pos | neg | neg | neg |  | pos | pos | pos | pos | pos | pos | pos | neg | pos | pos |  |
| MHK05027 | pos | pos | neg | neg |  | pos | pos | pos | pos | neg | neg | pos | neg | neg | neg |  |
| MHK05028 | pos | pos | neg | neg |  | neg | neg |  |  | pos | pos | pos | neg | pos | pos |  |
| MHK05042 | pos | pos | neg | neg |  | neg | neg |  |  | pos | pos | pos | neg | neg | neg |  |
| MHK05046 | pos | neg | neg | neg |  | neg | neg |  |  | neg | neg | neg | neg | neg | neg |  |
| MHK05068 | neg | pos | pos | neg | pos | neg | neg |  |  | pos | pos | pos | neg | neg | neg |  |
| MHK05070 | neg | neg | neg | neg |  | neg | neg |  |  | pos | pos | pos | neg | pos | pos |  |
| MHK05072 | pos | pos | neg | neg |  | pos | pos | pos | pos | pos | pos | pos | neg | pos | pos |  |
| MHK05080 | pos | pos | neg | neg |  | neg | neg |  |  | neg | neg | neg | neg | neg | neg |  |
| MHK05084 | neg | neg | neg | neg |  | pos | pos | pos |  | neg | neg | neg | neg | neg | neg |  |
| MHK05090 | neg | neg | neg | neg |  | neg | neg |  |  | neg | neg | neg | neg | neg | neg |  |
| MHK05091 | pos | pos | pos | neg | pos | pos | pos | pos | pos | pos | pos |  | neg | pos | neg |  |
| MHK05094 | neg | pos | neg | neg |  | neg | neg |  |  | neg | neg | neg | neg | neg | neg |  |
| NTS01697 | pos | pos | pos | neg | pos | neg | neg |  |  | neg | neg | pos | neg | neg | neg |  |
| NTS01699 | pos | pos | neg | neg |  | neg | neg |  |  | neg | neg | neg | neg | neg | neg |  |
| NTS01703 | pos | pos | neg | neg |  | neg | neg |  |  | neg | neg | neg | pos | pos | pos | pos |
| NTS01705 | pos | pos | neg | neg |  | pos | neg | pos | pos | pos | pos | pos | neg | neg | neg |  |
| NTS01707 | pos | neg | neg | neg |  | neg | neg |  |  | neg | neg | neg | neg | neg | neg |  |
| NTS01708 | pos | pos | neg | pos | pos | neg | neg |  |  | neg | neg | neg | neg | neg | neg |  |
| NTS01732 | pos | pos | neg | neg |  | neg | neg |  |  | neg | neg | neg | neg | neg | neg |  |
| NTS01745 | pos | pos | neg | neg |  | pos | neg | pos | pos | neg | neg | pos | neg | neg | neg |  |
| NTS01747 | neg | pos | neg | neg |  | pos | pos | pos | pos | neg | neg | neg | neg | neg | neg |  |
| NTS01749 | pos | pos | neg | neg |  | pos | pos | pos | pos | pos | pos | pos | neg | pos | pos |  |
| NTS01755 | pos | pos | neg | neg |  | pos | pos | pos | pos | pos | pos | pos | neg | pos | neg |  |
| NTS01793 | pos | pos | neg | neg |  | neg | neg |  |  | neg | neg | neg | neg | neg | neg |  |
| NTS01936 | pos | pos | neg | neg |  | neg | neg |  |  | neg | neg | neg | neg | neg | neg |  |

^a^Primer sequences given in Supplemental Table 2.

^b^sequences for the amplicons for the *E. coli*-derived *macAB* detected here have been submitted to NBCI under accession nos. NCBI accession nos. KX377891 through KX377893.

^c^pos — positive; neg — negative; blank spots indicate not done.
